# Missense Mutation in the Second RNA Binding Domain Reveals a Role for *Prkra* (PACT/RAX) during Skull Development

**DOI:** 10.1371/journal.pone.0028537

**Published:** 2011-12-14

**Authors:** Benjamin K. Dickerman, Christine L. White, Claire Chevalier, Valérie Nalesso, Cyril Charles, Sophie Fouchécourt, Florian Guillou, Laurent Viriot, Ganes C. Sen, Yann Hérault

**Affiliations:** 1 Department of Molecular Genetics, Lerner Research Institute, Cleveland Clinic, Cleveland, Ohio, United States of America; 2 Graduate Program in Molecular Virology, Case Western Reserve University, Cleveland, Ohio, United States of America; 3 Institut de Génétique Biologie Moléculaire et Cellulaire and Institut Clinique de la Souris, IGBMC/ICS, CNRS, INSERM, UMR7104, UMR964, Université de Strasbourg, Illkirch, France; 4 Team Evo-Devo of Vertebrate Dentition, Institut de Génomique Fonctionnelle de Lyon, Ecole Normale Supérieure de Lyon, CNRS, Université de Lyon, Lyon, France; 5 Physiologie de la Reproduction et des Comportements INRA/CNRS/Université de Tours/Haras Nationaux, UMR 6175 Centre de Recherche de Tours, Nouzilly, France; McMaster University, Canada

## Abstract

Random chemical mutagenesis of the mouse genome can causally connect genes to specific phenotypes. Using this approach, reduced pinna (rep) or microtia, a defect in ear development, was mapped to a small region of mouse chromosome 2. Sequencing of this region established co-segregation of the phenotype (*rep*) with a mutation in the *Prkra* gene, which encodes the protein PACT/RAX. Mice homozygous for the mutant *Prkra* allele had defects not only in ear development but also growth, craniofacial development and ovarian structure. The *rep* mutation was identified as a missense mutation (Serine 130 to Proline) that did not affect mRNA expression, however the steady state level of RAX protein was significantly lower in the brains of *rep* mice. The mutant protein, while normal in most biochemical functions, was unable to bind dsRNA. In addition, *rep* mice displayed altered morphology of the skull that was consistent with a targeted deletion of *Prkra* showing a contribution of the gene to craniofacial development. These observations identified a specific mutation that reduces steady-state levels of RAX protein and disrupts the dsRNA binding function of the protein, demonstrating the importance of the *Prkra* gene in various aspects of mouse development.

## Introduction

The *Prkra* gene encodes a double-stranded RNA binding protein, which was identified and named independently as Protein Activator of PKR (PACT) in human [1], and PKR-associated protein X (RAX) in mouse [2]. PACT and RAX are almost identical in their amino acid sequences; only 6 out of 313 residues are different with 4 substitutions being with similar residues. Initial studies on this protein were focused on its ability to induce autophosphorylation of and activate interferon inducible, double-stranded RNA dependent protein kinase (PKR) (encoded by the *Eif2ak2* gene) in response to various stresses such as ceramide [3], arsenite [2], [4], tumor necrosis factor α (TNFα) [5], ethanol [6], low dose actinomycin D [7], growth factor withdrawal [2], [4], chemotherapeutics [8], endoplasmic reticulum (ER) stress [9], [10], or peroxide [2], [4]. Activation of PKR results in phosphorylation of eukaryotic initiation factor 2α (eIF2α) leading to inhibition of protein synthesis [11], [12]. In addition to PACT/RAX, PKR is modulated by another dsRNA binding protein, TAR (*trans*-activating region) RNA-binding protein (TRBP in human, PRBP in mouse) (encoded by the *Tarbp2* gene) [13], [14], [15]. In contrast to PACT/RAX, TRBP/PRBP inhibits PKR activation [14]. Aside from binding PKR, PACT and TRBP have also been shown to heterodimerize through interaction of their N-terminal dsRNA binding motifs, as well as through their C-terminal Merlin-Dicer-PACT liaison (Medipal) domain [16], [17].

Upon appropriate stimulation, PACT is phosphorylated on serine 246 and serine 287 [18], while RAX is phosphorylated on serine 18 [19]. Phosphorylation causes PACT/TRBP heterodimers to dissociate [20], [21], freeing PACT to bind PKR through its two amino-terminal double stranded RNA binding domains [7]. This leads to conformational change facilitating interaction of PACT's carboxy-terminal domain with the kinase domain of PKR (residues 328–335) leading to PKR activation [16], [22], [23] and subsequent eIF2α activation.

Studies in mice in which the *Prkra* gene was disrupted (*Prkra^tm1Gsc/tm1Gsc^* mice) produced unexpected results. In contrast to mice in which the *Eif2ak2* gene has been disrupted (*Eif2ak2^tm1Cwe/tm1Cwe^*), which had no discernable developmental phenotype [24], *Prkra^tm1Gsc/tm1Gsc^* mice showed defects in ear and craniofacial development, growth and fertility [25]. Further investigation revealed that *Prkra^tm1Gsc/tm1Gsc^* mice developed hypoplastic anterior pituitaries resulting from reduced cell proliferation in this tissue [26]. As the anterior pituitary contains cells which secrete hormones required for growth and sexual development, this likely accounts for some of the developmental anomalies observed in the mouse [26].

In addition to its ability to activate PKR, PACT has also been shown to have a role in production of small RNAs involved in RNA silencing. PACT (as well as TRBP) interacts with Dicer, which processes small RNAs from their precursor to mature forms, and is a component of the RNA Induced Silencing Complex (RISC) whose key components include Dicer and Argonaut proteins [27]. While not essential for cleavage of pre-microRNAs to their mature form by Dicer, PACT may be required for RISC assembly, as depleting PACT led to reduced levels of mature miRNAs *in vitro* [27]. While knockout mice for Dicer have been generated, these are embryonically lethal [28]. The relevant tissue specific knockouts for Dicer, however, [29], [30] show similar reproductive defects to the *Prkra^tm1Gsc/tm1Gsc^* mice. This observation supports the idea that at least some of the developmental defects seen in the *Prkra* deficient mouse might result from defects in miRNA processing.

In humans, mutations in *Prkra* are associated with Dyt16, an autosomal recessive young onset dystonia-parkinsonism disorder [31], [32]. Dyt16 patients show retarded speech learning in infancy and involuntary muscle contraction starting during teenage years. The respective mutations correspond to a frameshift (266–267delAT) causing premature termination of the protein [32] and to a missense mutation P222L [31].

Ethylnitrosylurea (ENU) mutagenesis provides a mechanism for generating random point mutations in the mouse germline [33]. Offspring of mice that have been mutagenized can be sequentially crossed with wild-type mice to segregate mutated recessive alleles and intercrossed to generate mice homozygous for the recessive mutation [34]. The location of a mutation can then be mapped within the genome and the mutated gene can be identified [34], [35]. Subsequent to performing such a screen for dysmorphology one mouse line named *rep* for “reduced pinna” was identified and established carrying a recessive mutation in *Prkra*.

This study describes similarities and differences in the phenotypes of the *rep* mutant mouse generated by ENU mutagenesis and the existing *Prkra* null mutant mouse (*Prkra^tm1Gsc/tm1Gsc^*, described above) in which a portion of Exon 8 of *Prkra* was replaced with a neomycin resistance cassette [25]. This study demonstrates that the *rep* mutation produces a mutant of the PACT/RAX protein which is present at significantly reduced steady-state levels. This low level of PACT/RAX results in a phenotype with many similarities to that of the *Prkra^tm1Gsctm1Gsc^* mouse in which no protein can be detected, in contrast to the lethal effect observed in the deletion of the entire gene [36].

## Results

### Characterization of the reduced pinna recessive mutation affecting the *Prkra* gene

**Figure 1:**
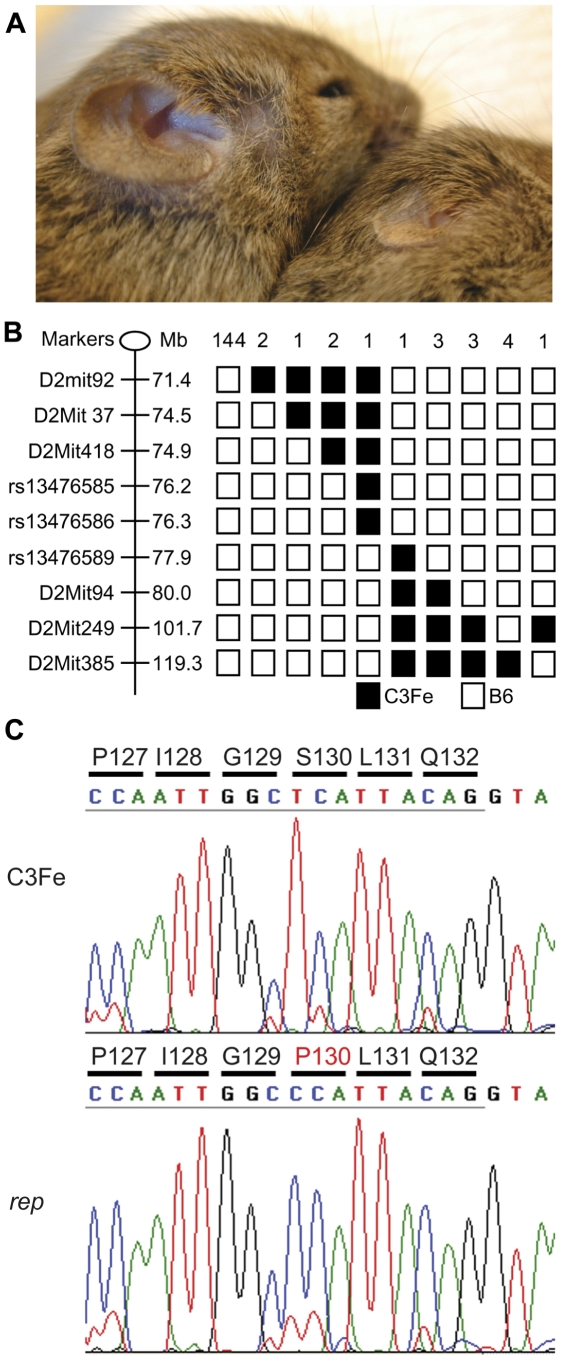
Characterization and genetic identification of the *rep* mutation. ***A*** 15days post-partum *rep* homozygous mice (right) displayed reduced pinna compared to control littermates (left). ***B*** Haplotype analysis of 81 mutant mice derived from the outcross-intercross strategy. Markers are shown with their position (cM) on chromosome 2 (Ensembl V50). The C57BL/6J alleles are shown with black boxes whereas the white boxes indicate the presence of the C3HeB/FeJ allele. Haplotypes with the same allelic distribution were collected and their number is given at the top of each column. ***C*** Sequence chromatograph spanning the *rep* mutation site compared with that of a wild-type control.

**Figure 2:**
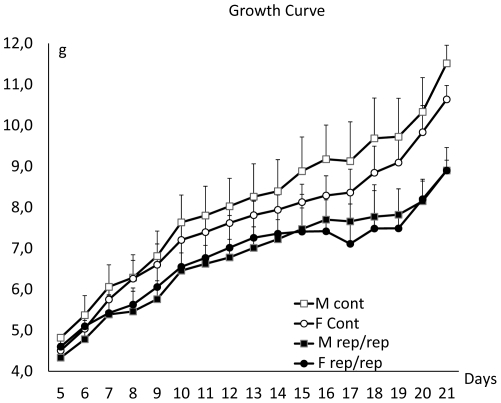
Growth curves of the *rep* homozygote mutants and control littermates. Body weight curves of *rep/rep* individuals generated from heterozygote intercrosses (male *rep/rep* n = 9; male wt n = 15; female *rep/rep* n = 12; female wt n = 22) reveal that growth of mutant homozygotes is affected during post-natal development compared to control littermates.

**Figure 3:**
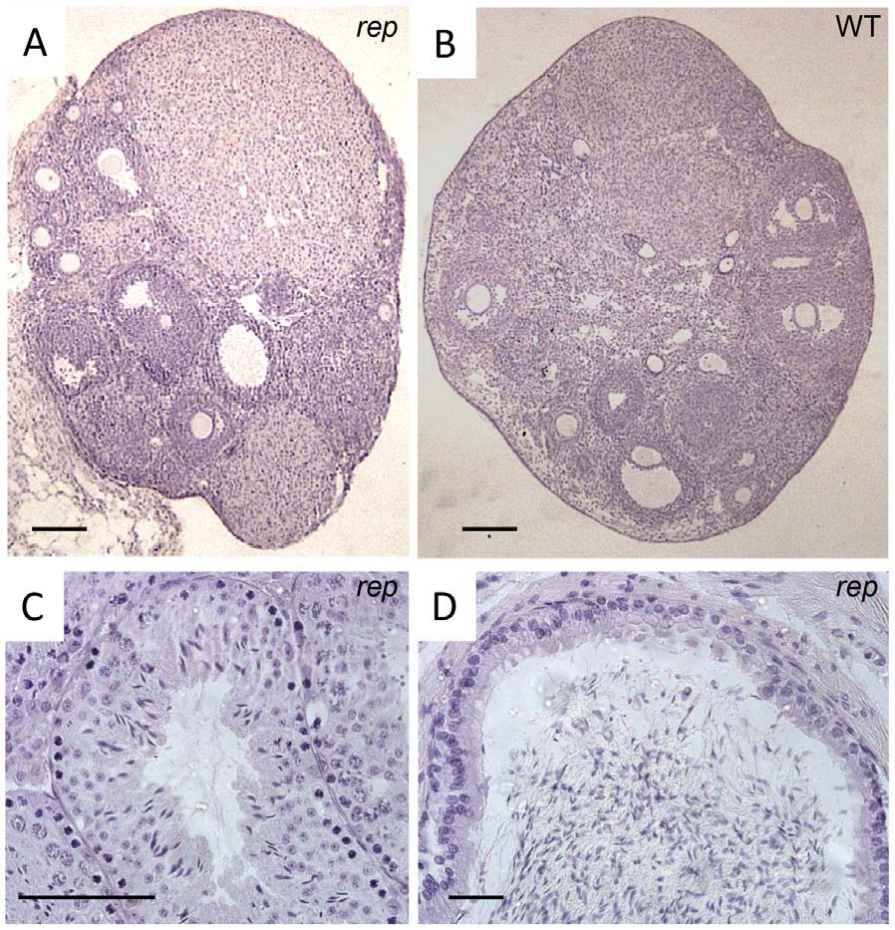
Analysis of the gonads of *rep/rep* mutant mice. ***A, B*** Histological analysis of hematoxylin-stained sections. In ovaries, all stages of folliculogenesis from primordial to preovulatory follicles and corpus luteum were observed in mutant mice (***A***) as in wild-type (***B***), suggesting that ovaries are functional. Scale bar = 150 µm. ***C*** Histology of the testis from adult *rep* mutant male. All stages of spermatogenesis were visible, and no alteration of tubule diameter was observed. Scale bar = 50 µm. ***D*** Histology of the cauda epididymis from adult *rep* mutant male. No abnormalities were observed. Scale bar = 20 µm.

**Figure 4:**
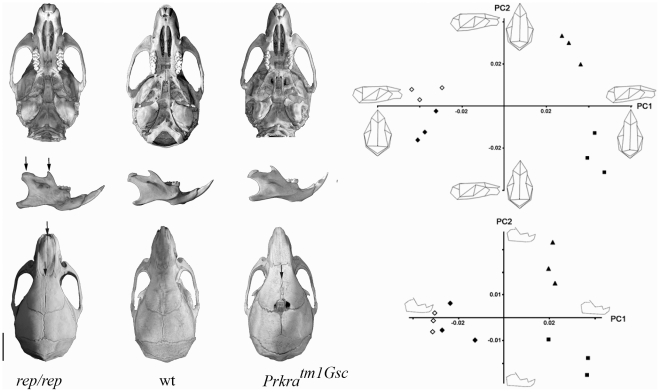
Morphological variations observed in skulls and mandibles of wt, *rep* and *Tm1Gsc* mice. ***A*** Major cranial anomalies observed in *Prkra^rep^* and *Prkra^tm1Gsc^* mice. Arrows indicate the principal defects for each mutant, *i.e.* the reduced coronoid process and mandibular condyle, and the short nasal bone in *rep* mice and the bregmatic fontanelle-like structure of *Prkra^tm1Gsc^* mice. Scale bar: 5 mm. ***B*** Plot of principal components 1 and 2 based on Procrustes analysis of 3D landmark coordinates of wt, *rep* and *Prkra^tm1Gsc^* mice. Squares: wt mice; Triangles: *Prkra^tm1Gsc^* mice; Filled diamonds: *rep* heterozygous mice; Open diamonds: *rep* homozygous mice. On the principal components analysis (PCA) performed from cranial landmarks (top panel), PC1 represents 54.7% of variance and PC2 15.1%. On the PCA performed from mandibular landmarks (bottom panel), PC1 represents 46.0% of variance and PC2 16.1%.

In the course of the Phenotype Homozygote Mutants program [34], the *rep* mutant mouse line which displays microtia (Figure 1A) and growth retardation (Figure 2, Table 1) was isolated. The mutation was induced by ENU on the C57BL/6J background and was further established by backcrossing on the C3HeB/FeJ genetic background. We used this backcross to precisely map the position of the mutation using a panel of markers already described [34], [35]. The *rep* mutation was located between rs13476586 and rs13476589 on mouse chromosome 2 (Figure 1B). Looking at candidate genes in this region, we identified the *Prkra* gene for which a knock-out displaying similar dysmorphology was previously described [25]. We sequenced the *Prkra* coding sequence and exon/intron borders. We found a point mutation T→C in exon 4 affecting codon 130 and introducing the missense mutation S130P (Figure 1C). Thus we conclude that the *rep* mutation affected the *Prkra* gene so we named the mutation *Prkra^rep^*. We verified that this change was not found in either C57BL/6J or C3HeB/FeJ background, or in other mouse strains including 129S2, BALB/c and DBA2/J (data not shown). Performing a more detailed phenotypic analysis we noticed two major changes in *Prkra^rep/rep^* homozygous mice. First, adult homozygous mice were smaller compared to their control littermates (Table 1). The weight difference was also found in younger individuals starting at 7 days post partum, with no major difference between sexes in homozygous mice (Figure 2). Second we noticed a defect in fertility when we crossed the *Prkra^rep/rep^* females with wild-type (wt) or heterozygous mice. No progeny were obtained by breeding 7 homozygous females with wt males over a 2 month period. Histopathological analysis of the ovaries showed all stages of folliculogenesis from primordial to preovulatory follicles and corpus luteum formation were present in mutant mice (Figure 3A, B). No abnormalities were observed in the histology of testes from male mutants (Figure 3C, D). We also observed differences in the skull of *rep* homozygous mutants. We characterized those changes by 3D-cranial morphology assessment using a series of landmarks as indicated in the Materials and Methods. *Prkra^rep/+^* (data not shown) and *Prkra^rep/rep^* mouse skulls were characterized by very short nasal bones (Figure 4A). They also differed from the wt morphology by the shape of the zygomatic process of the temporal bone which was relatively more robust than the wt and their mandible differed from the wt by a reduced coronoid process and a reduced mandibular condyle (Figure 4A). Interestingly, *Prkra^tm1Gsc/tm1Gsc^*specimens presented an open area at the top of the skulls resulting from the lack of fusion of the frontal and parietal bones. They also differed from the wt morphology by a reduction of the interparietal bone associated with a medial displacement of the parietal/interparietal/occipital junction. Mandibles of homozygous specimens presented a reduced mandibular condyle when compared to the wt specimens. The cranial and mandibular morphologies of *Prkra^rep/+^* (data not shown), *Prkra^rep/rep^* and *Prkra^tm1Gsc/tm1Gsc^* individuals differed from the wt and also differed from one another (Figure 4B). These data highlight that although phenotypes resulting from the *Prkra^rep^* and *Prkra^tm1Gsc^* mutations share many common features, the mutations have different impacts on cranial and mandibular morphology.

**Table 1:** Weights of adult *rep* mice compared to those of WT controls^a^.

Sex	WT	*rep/rep*	P Value
Male	24.9+/−2.1 gn = 11	19.2+/−2.4 gn = 10	0.0013
Female	21.8+/−1.1 gn = 8	17.5+/−0.5 gn = 4	0.0005

a Weights of adult *rep* and WT littermates (male age 54 days+/−1.4 days; female age 54.3+/−1.3 days) and the P-values of the corresponding Student T-test.

### Presence of RAX mRNA in the brains of *rep* mice

**Figure 5:**
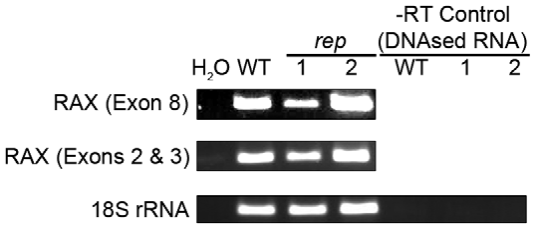
Analysis of RAX mRNA expression in the brains of *rep* mice. Total RNA was isolated from one WT and two *rep* mouse brains, treated with DNase and analyzed by RT-PCR with appropriate primers to measure the levels of the 5′ region (exons 1 and 2) and the 3′ region (exons 7 and 8) of RAX mRNA. 18S rRNA was measured as a loading control and –RT controls were used to ensure the absence of any genomic DNA in the RNA preparations.

To understand the physiological defects seen in *rep* mice it was important to determine whether *Prkra* mRNA was present at wild-type levels in these animals. It was possible that the S130P mutation would alter the production, processing or stability of *Prkra* mRNA. RT-PCR was used to determine whether *Prkra* mRNA was produced in brain. Primer sequences targeting the 5′ region (exons 2 and 3) and 3′ region (exon 8) were used in separate reactions to determine whether the entire mRNA or simply the portion 5′ of the T-C substitution leading to the S130P mutation was generated. Both 5′ and 3′ portions of *Prkra* mRNA were present in *rep* mice at levels comparable to those seen in wt mice, indicating the S130P mutation does not impair production of full length *Prkra* mRNA (Figure 5).

### RAX (S130P) dimerizes and activates PKR in response to stress, but is unable to bind dsRNA

To investigate the biochemical basis for the *rep* phenotype, the S130P mutation was introduced in RAX for expression in mouse and bacterial cells. The mutant protein was characterized for its ability to bind and activate PKR, bind dsRNA and homodimerize.

**Figure 6:**
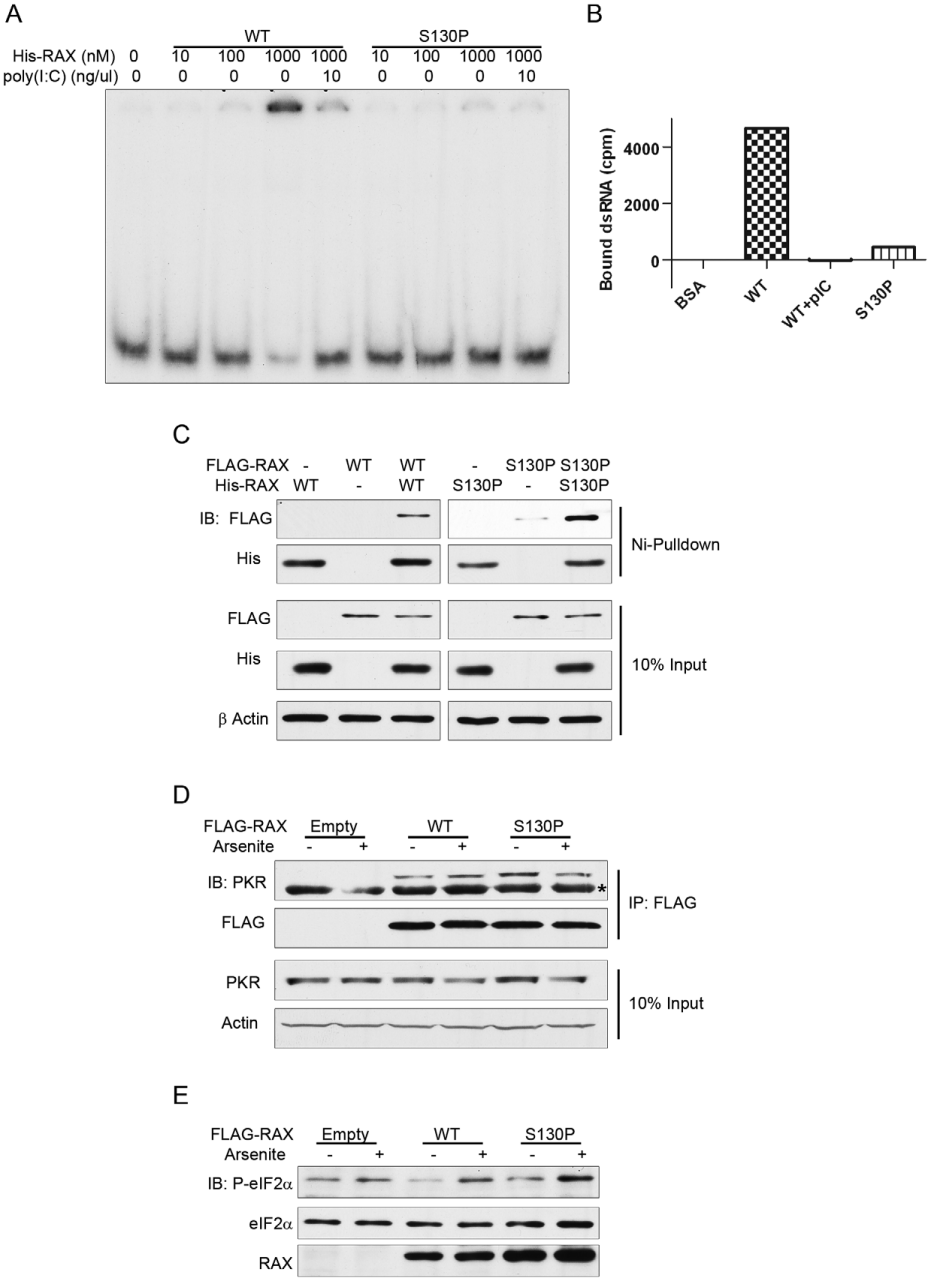
Functional characterization of the RAX (S130P) mutant. ***A*** **DsRNA electrophoretic mobility shift assay:** Purified His-RAX or His-RAX (S130P) was incubated at the indicated concentration with 5′end labelled dsRNA. In the indicated lanes, poly(I:C) was added to the reaction as a competitor to demonstrate dsRNA-binding specificity. ***B*** **DsRNA-pull-down assay:** Purified His-RAX or His-RAX (S130P) was incubated with 5′end labelled dsRNA. His-RAX was pulled-down from the reactions with Ni-NTA agarose and bound dsRNA was measured by liquid scintillation (expressed in counts per minute). CPM bound to BSA control has been subtracted from all lanes as background. ***C*** **Dimerization assay:** Purified His-RAX or His-RAX (S130P) was incubated with lysates from L929 cells expressing empty provirus, FLAG-RAX or FLAG-RAX (S130P). His-RAX was pulled-down from the reactions with Ni-NTA agarose and incubated with micrococcal nuclease to eliminate dsRNA-facilitated protein-protein interactions. Samples were resolved by SDS-PAGE and subjected to western blotting with the indicated antibodies. Total input lysates were directly analyzed for measuring the levels of expression of the indicated proteins. ***D*** **PKR interaction assay:** FLAG-RAX was immunoprecipitated with an agarose-conjugated antibody against FLAG from lysates prepared from unstressed or arsenite treated L929 cells expressing empty provirus, FLAG-RAX or FLAG-RAX (S130P). Immunoprecipitated samples were incubated with micrococcal nuclease to eliminate dsRNA-facilitated protein-protein interactions. Samples were resolved by SDS-PAGE and PKR was detected by western blot to measure RAX-PKR interaction (top panel: PKR is the upper band and IgG is the lower band (denoted by *)). Other panels show the levels of FLAG-RAX in the immunoprecipitates and the levels of PKR and actin in the input lysates. ***E*** **PKR activation assay:** eIF2α phosphorylation was monitored by western blotting with a phospho-eIF2α-specific antibody (top panel). The middle and the bottom panels show the levels of eIF2α and RAX respectively. In these experiments, expression of endogenous RAX had been ablated by expressing a shRNA directed toward the 3′UTR of the mRNA. In those cells, WT or mutant RAX was ectopically expressed using lentivirus vectors; cells were stressed by treating them with sodium arsenite (100 µM), where indicated.

The ability of RAX (S130P) to bind dsRNA was determined by electrophoretic mobility shift assays using radiolabeled dsRNA and bacterially-expressed, purified WT and mutant His-RAX. There was a clear shift in electrophoretic mobility of the dsRNA probe in the presence of 1 µM WT His-RAX which could be competed out by unlabeled synthetic dsRNA, poly(I:C), demonstrating dsRNA specific binding activity of WT RAX. The mutant protein however could not bind dsRNA, as measured by this assay (Figure 6A). To further examine this observation, a different dsRNA-binding assay was used. In this assay, His-RAX was incubated with radiolabeled dsRNA and then purified using Ni-NTA agarose; the amount of dsRNA probe, bound to RAX, was quantified by scintillation counting. Again, there was clear dsRNA binding to the WT protein, which could be competed out with the addition of unlabeled poly(I:C), while the mutant protein was unable to bind the dsRNA probe (Figure 6B). These results demonstrate that the S130P mutation disrupts the dsRNA binding capacity of RAX.

Dimerization was examined by incubating purified WT or S130P His-RAX with lysates from L929 cell lines expressing similar levels of WT or S130P FLAG- RAX, as generated by lentiviral transduction (Figure 6C, middle panels). Both WT and mutant RAX homodimerized (Figure 6C, top panel) demonstrating that the mutation does not interfere with dimerization of RAX.

To assess the ability of the S130P mutant protein to bind PKR, cell lysates from WT or S130P FLAG-RAX expressing L929 cells treated with sodium arsenite (to induce RAX phosphorylation) were immunoprecipitated using a FLAG antibody, followed by western blot for endogenous PKR. There were clear interactions between PKR and WT or S130P RAX proteins, independent of the arsenite treatment (Figure 6D), demonstrating that the S130P mutation does not interfere with PKR interaction of RAX.

For testing the ability of RAX (S130P) to activate PKR, we generated L929 cells in which expression of RAX had been ablated by a shRNA that targets the 3′UTR of RAX mRNA. In this cell line, WT or mutant RAX was ectopically expressed using lentiviral vectors encoding the corresponding RAX mRNAs without the UTRs (Figure 6E, bottom panel). These cells were treated with sodium arsenite to activate RAX whose ability to activate PKR was monitored by measuring eIF2α phosphorylation; there were comparable stress-induced increases in phospho-eIF2α in cells expressing WT or mutant RAX (Figure 6E, top panel) demonstrating that the S130P mutation does not impair the stress-induced PKR activation function of RAX.

### Reduced steady-state levels of ectopically expressed mutant RAX

**Figure 7:**
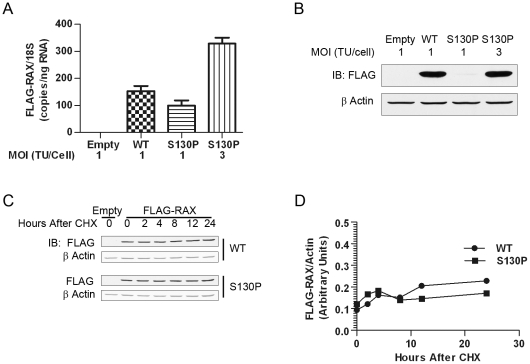
Reduced ectopic expression of RAX (S130P) in L929 cells. ***A*** **RAX mRNA levels:** Realtime RT-PCR analyses were used to determine the levels of FLAG-RAX mRNA relative to 18S rRNA using RNA samples isolated from the indicated cells. ***B*** **RAX protein levels:** FLAG western blot of L929 cells infected with empty lentivirus, or lentivirus encoding a provirus to ectopically express FLAG-RAX or FLAG-RAX (S130P) at the indicated MOI. Three times more virus was required for FLAG-RAX (S130P) to achieve protein levels comparable to WT. ***C*** **RAX turnover analyses:** Cells were treated with cycloheximide to inhibit *de novo* protein synthesis, cells lysates were prepared at the indicated time points and protein levels were measured by Odyssey quantitative western blot of FLAG- RAX and FLAG-RAX (S130P) using actin as the internal control. ***D*** **Normalized levels of RAX:** FLAG-RAX signal was normalized to that of actin and plotted at the indicated times following cycloheximide treatment.

In L929 cells, ectopic expression levels of FLAG-RAX (S130P) were consistently lower than those of WT FLAG-RAX (data not shown). To determine whether the observed difference was operative at the transcription or the translation level, we measured the levels of RAX protein by western blot and mRNA by realtime RT-PCR, in several cells lines that we generated. In cells infected with RAX-expressing lentivirus at moi of 1, as compared to WT, there was a slightly reduced level of the mutant mRNA (Figure 7A) but an almost undectable level of the mutant protein (Figure 7B). However, when the mutant-expressing virus was used at a moi of 3, a comparable level of the mutant protein was expressed from more than twice the level of the mutant mRNA, indicating a defect in the synthesis or turnover of the mutant protein. To distinguish between the two possibilities, we measured the stability of the mutant protein in cells expressing equal levels of WT and mutant proteins. New protein synthesis was inhibited by cyclohexamide treatment, and the rate of decay of existing RAX was monitored by western blot analysis at different time points (Figure 7C). Data were quantified as FLAG signals relative to actin signals (Figure 7D). We did not observe any significant difference in the rates of WT and mutant protein decay. These results indicate that the lower steady-state level of the mutant protein is probably due a defect in its synthesis, but not due to an accelerated decay.

### Significantly reduced levels of mutant RAX in the brains of *rep* mice

**Figure 8:**
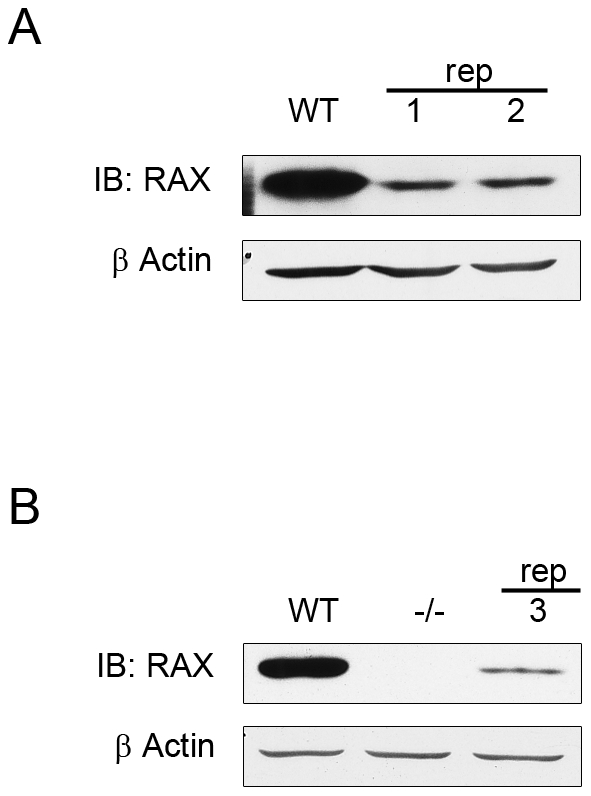
Lower levels of RAX protein in the brains of *rep* mice. ***A*** **RAX levels in proteins recovered from the same samples used for RAX mRNA measurements:** Protein re-precipitated from trizol extracts of the same brain preparations shown in Figure 5 was analyzed by western blot for RAX and actin expression. ***B*** **RAX levels in conventional protein extracts of brains:** RAX western blot from brains homogenized and lysed in detergent-lysis buffer (see materials and methods). RAX expression was analyzed in wild-type, *Prkra^tm1Gsc^* (denoted −/−) and *rep* (denoted 3) mice.

To extend our observations in cell lines to mice, we measured RAX protein levels in brains of WT and *rep* mice; we chose the brain as a representative tissue because RAX is highly expressed there. Proteins precipitated from the trizol extracts of the brains of the mice used to measure RAX mRNA levels (Figure 5) was used to detect RAX protein levels by western blot, revealing significantly reduced levels in mutant mice compared with WT mouse (Figure 8A). To rule out the possibility that the reduced protein levels were an artifact of precipitation from the trizol fractionation, we prepared a third *rep* brain, along with WT and *tm1Gsc (RAX−/−)* brains, using conventional detergent lysis. These additional samples showed reduced mutant RAX protein in the *rep* mouse and no RAX in the RAX−/− mouse brains (Figure 8B). These results, combined with those shown in Figure 5, indicate that in *rep* mice much less RAX is present because of a defect in the synthesis of the mutant protein.

## Discussion

In this report we described the characterization of a new ENU-induced missense mutation (S130P) in the coding sequence of the *Prkra* gene. The new allele of the *Prkra* gene (*rep*) induces several alterations in growth, and the development of the ear and skull of the mutant mice. During this analysis we further characterized in *rep* homozygous mutants, the craniofacial defects previously observed in *tm1Gsc* mice. *Rep* mice were found to have a very short nasal bone and a reduced coronoid process and mandibular condyle. Parallel analysis carried out for the *Prkra^tm1Gsc^* mutation revealed similar mandibular alteration but major changes of the skull shape with a lack of fusion of the frontal and parietal bones. These series of data elaborate upon the role of *Prkra* in controlling cranio-facial development.

The S130P mutation is located in the second dsRNA binding domain of the protein and disrupts dsRNA binding without affecting RAX dimerization or its ability to activate PKR. The mutation also leads to a decrease in steady-state levels of RAX protein, in tissue such as the brain, resulting in the phenotypes observed in the *rep* mutant which are similar to those of a mouse with a targeted disruption in the *Prkra* gene and as such the phenotype is likely a result of the significantly reduced steady-state protein level. At this time, we can not discern whether this is connected to the inability of the mutant protein to bind dsRNA. Another factor, that may play a role, is TAR RNA binding protein (TRBP); its binding to PACT is partly mediated by the second domain, the site of the S130P mutation [17].

Human TRBP and PACT directly interact with each other and associate with Dicer to facilitate the production of small interfering RNA [27], [37]. Recently, Zehir *et al.* (2010) demonstrated that controlled inactivation of *Dicer* in neural crest cells (which function in skull development) results in craniofacial malformation [38]. The phenotypes we have observed in *Prkra* mutant mice may be reminiscent of hypomorphic phenotypes of Dicer indicating that defects in the miRNA pathway might contribute to the craniofacial malformations we observed. As the *rep* mutation disrupts dsRNA binding by RAX, there may be an effect on the miRNA pathway in the *rep* mutant.

The *rep* mutant is, to our knowledge, the third allele of *Prkra*. Rowe *et al.* (2006) published the *Prkra^tm1Gsc^* mutant for *Prkra* with a targeted disruption of the 3′end of the gene that was used here for comparison. Almost all the phenotypes present in the *tm1Gsc mouse* were recapitulated to some extent in the *rep* mutant. Interestingly the two mutations respectively affect the second and the third RNA binding domains of the protein. In contrast, deletion of the entire gene, *Prkra^tm1Wsmay^*, engineered by Bennett *et al.* (2008), induces an early developmental lethality with no embryos detected after 3.5 days post conception. The less severe phenotype seen in the *Prkra^tm1Gsc^* and *Prkra^rep^* mutants could occur because these mutant proteins retain some function mediated by the domains that have not been mutated. Indeed, although the protein encoded by the *Prkra^rep^* allele is deficient in dsRNA binding, it still maintains dimerization activity and the ability to activate PKR. Similarly, the *Prkra^tm1Gsc^* transcript could potentially be translated, although it contains multiple stop codons in all 3 reading frames and has never yielded detectable protein [25]. The mutant protein identified in *rep* and theoretically present in *tm1Gsc* shared similar properties and activities suggesting that a minimal level of expression of the RAX protein, as observed in the *rep* homozygote animal, is necessary and sufficient to rescue the early embryonic phenotypes observed in *Prkra^tm1Wsmay^* mutant.

The *Prkra^tm1Gsc^* and *Prkra^rep^* mutants represent a class of hypomorphic allele with similar phenotypes affecting growth, ear, craniofacial development, fertility and pituitary hormone production (not shown). They should be considered as good candidate models for studying Dystonia 16 (Dyt16) which is linked to mutations in the human *PRKRA* gene leading to either truncated form of the protein or changes in amino-acid sequences. So far two mutations have been described in Dyt16 patients that correspond to a frameshift (266–267delAT) causing premature termination of the protein [32], and to a missense mutation P222L [31]. These complementary data suggest that the strong phenotype observed in the *Prkra^tm1Wsmay^* allele is more a consequence of the complete absence of RAX protein with no residual activities rather than the inactivation of the first binding domain. Until now the *Prkra* partial loss of function mutants have been studied for other purposes but both models should be explored further for traits observed in human patients to be validated as a model for Dystonia 16.

## Materials and Methods

### Mice

The *rep* mutation was isolated from PhenHomut a genome wide recessive mutagenesis program for phenotyping homozygote mutants previously described [34], [35]. The screening was oriented toward recessive mutations affecting morphology, the cardiovascular system, metabolism and the immune response. For *rep*, F1 males, derived from the first progeny of ENU-treated C57BL/6J (B6) males, were mated with wild-type females C3HeB/FeJ (C3H) to generate G2 individuals. G3 individuals were derived from the backcross of G2 females with the F1 males, and were screened following a hierarchical and standardized phenotyping analysis. We also took advantage of the backcross for the genetic mapping and phenotypic analysis as described previously [34], [35]. For analysis of RAX expression, brains were dissected, snap-frozen and stored in liquid nitrogen prior to isolation of RNA and/or protein. All experiments were performed within the guidelines of the French Ministry of Agriculture for experiments with laboratory animals or in strict accordance with the recommendations in the Guide for the Care and Use of Laboratory Animals of the National Institutes of Health. The protocols were approved by the Institutional Animal Care and Use Committee of Cleveland Clinic (Approval Number ARC 08738) or the Ethical Committee for the Region Centre to Y Herault (law 87 848; YH accreditation 45–31). All efforts were made to maximize animal welfare.

### Phenotypic analysis of mice

Cohorts of age and sex-matched mutant and wild-type mice were tested for a variety of phenotypic parameters according to the standard operating procedures of the EUmorphia network [34], [35]. Mice were weighed daily to generate growth curves. For histological analysis of gonads, tissues were fixed in Bouin's fixative, embedded in paraffin, sectioned at a thickness of 7 µm and stained with hematoxylin. For X-ray analysis skulls were preserved in 95% ethanol at 4°C prior to analysis.

To examine variations in skull and mandible shape, we used a 3D morphometric method based on landmark comparisons adapted from Hallgrimsson *et al.* 2004 [39]. Landmarks were taken on virtual reconstructions of the specimens' skulls and mandibles obtained by X-ray microtomography. The resulting voxel size varied among specimens between 18 and 24 µm. The skull and mandible surfaces were extracted using VGStudiomax software. Twenty-two landmarks were defined using Landmark software on each mandible and forty-nine on each skull (Figure S1). Specimen size was normalised and landmarks from different specimens superpimposed using the Procrustes method. Principal Component Analyses was performed using Morphologika^2^ v2.4 software using the full tangent space projection. Statistical tests (MANOVA) were performed using Statistica software.

### Plasmids, cell Lines and reagents

Mouse L929 and human HT1080 and HEK293T cell lines were cultured in Dulbecco's modified Eagle's medium containing 10% fetal bovine serum (Atlanta Biologicals) supplemented with glucose (4.5 g/L), penicillin (50 U/ml), streptomycin (50 µg/ml), L-glutamine (2 mM) and sodium pyruvate (1 mM). Sodium arsenite was obtained from Sigma. Trizol reagent was obtained from Invitrogen. The plasmids pLVX-IRES-ZsGreen1 and pVSG-G were obtained from Clontech, pCMV-R8.74 was obtained from Addgene, pET15b was obtained from Novagen, pcDNA3 was obtained from Invitrogen, pLKO.1-puro was obtained from Sigma.

### Cloning of FLAG-RAX

The oligonucleotides AGC TTG GTA CCA TGG ACT ACA AGG ACG ATG ACG ATA AGC ATG and GAT CCA TGC TTA TCG TCA TCG TCC TTG TAG TCC ATG GTA CCA were annealed and ligated into HindIII and BamHI digested pcDNA3 (Invitrogen) using Rapid DNA Ligation Kit (Roche) to generate pcDNA3-FLAG. RNA was extracted from L929 cells using Trizol (Invitrogen) according to the manufacturer's instructions, followed by DNAse treatment using DNA-free (Ambion) according to manufacturer's instructions. Extracted, DNase-treated RNA was then reverse transcribed with the Superscript III system (Invitrogen) using random hexamers according to manufacturers instructions. The resulting cDNA was used to PCR the coding sequence of RAX using the primers: 5′ AGT AGT GGA TCC ATG TCC CAT AGC AGG C and 3′ AGT AGT TCT AGA CTA CTT TCT TTC TGC TAT TAT C with Expand High Fidelity PCR System (Roche), the PCR product was then digested with BamHI and XbaI (New England Biolabs) and ligated into BamHI-XbaI digested pcDNA3-FLAG with Rapid DNA Ligation Kit (Roche), this clone was designated pcDNA3-FLAG-RAX. pcDNA3-FLAG-RAX was used as a template in a PCR reaction using the primers: 5′ 5′AAC TCG AGG TAC CAT GGA CTA CTA CAA GG and 3′ AAT CTA GAG GAT CCC TAC TTT CTT TCT GCT ATT ATC TTT AAA T with Expand High Fidelity PCR System (Roche), the product was digested with XhoI and XbaI (New England Biolabs) and ligated into pLVX-IRES-ZsGreen1 (Clontech) with Rapid DNA Ligation Kit (Roche) to generate pLVX-FLAG-RAX-IRES-ZsGreen1.

### Mutation of FLAG-RAX

The S130P mutation was introduced to pLVX-FLAG-RAX-IRES-ZsGreen1 by primer overlap mutagenesis, briefly one PCR reaction was performed using the primers: 5′AAC TCG AGG TAC CAT GGA CTA CTA CAA GG and 3′ GCT AAT TCC TGT AAT GGG CCA ATT GGA TTC AGC TGG while a second PCR reaction was performed using the primers: 5′ CCA ATT GGC CCA TTA CAG GAA TTA GCA ATT CAC CAT G and 3′ AAT CTA GAG GAT CCC TAC TTT CTT TCT GCT ATT ATC TTT AAA T, both reactions were performed using Expand High Fidelity PCR System (Roche). The resulting overlapping PCR products were then used as template for a second round PCR using the primers: 5′ AAC TCG AGG TAC CAT GGA CTA CTA CAA GG and 3′ AAT CTA GAG GAT CCC TAC TTT CTT TCT GCT ATT ATC TTT AAA T with Expand High Fidelity PCR System (Roche), the mutagenized product was digested with XhoI and XbaI (New England Biolabs) and ligated into pLVX-IRES-ZsGreen1 (Clontech) with Rapid DNA Ligation Kit (Roche) to generate pLVX-FLAG-RAX(S130P)-IRES-ZsGreen1.

### Construction of the RAX shRNA expression clone

The DNA oligonucleotides CCG GCC GTC AAC TTT CCA GAT TTC TCG AGA AAT CTG GAA AGT TGA CGG TTT TTG and AAT TCA AAA ACC GTC AAC TTT CCA GAT TTC TCG AGA AAT CTG GAA AGT TGA CGG were annealed and ligated into pLKO.1-puro digested with AgeI and EcoRI (New England Biolabs) using Rapid DNA Ligation Kit (Roche) to generate pLKO.1-RAX (1199)-puro.

### Production of and infection with recombinant lentiviruses

The lentiviral plasmids pLVX-IRES-ZsGreen1, pLVX-FLAG-RAX-IRES-ZsGreen1, pLVX-FLAG-RAX (S130P)-IRES-ZsGreen1 or pLKO.1-RAX (1199)-puro were cotransfected with the packaging plasmid pCMV-dR8.74 and the pseudotyping plasmid pVSV-G by calcium phosphate into HEK293T cells. Lentivirus-containing supernatants were harvested three times every 12–16 hours, the collections were pooled, and the lentivirus titered on HT1080 cells by estimating percentage ZsGreen1 positive cells microscopically. L929 cells were infected with recombinant lentivirus in complete DMEM (containing 10% FBS, penicillin and streptomycin) containing 8 µg/ml polybrene (hexadimethrine bromide) for 24 hours before splitting into fresh media without polybrene and passaging as a stable line. For RAX knockdown with pLKO.1-RAX (1199)-puro, after 48 hours of recovery in complete DMEM, puromycin was added at 5 µg/ml; after selection the cells were grown in puromycin containing media as a stable line.

### Bacterial expression and purification of recombinant RAX

pLVX-FLAG-RAX-IRES-ZsGreen1 was used as a template in a PCR using the primers: 5′ AAA AGC TTG GAT CCT ATG TCC CAT AGC AGG CAT CG and 3′ AAT CTA GAG GAT CCC TAC TTT CTT TCT GCT ATT ATC TTT AAA T using Expand High Fidelity PCR System (Roche), the product was digested with BamHI (New England Biolabs) and ligated into pET15b (Novagen) using Rapid DNA Ligation Kit (Roche) to generate pET15b-RAX. pLVX-FLAG-RAX(S130P)-IRES-ZsGreen1 was used as a template in a PCR using the primers: 5′ AAA AGC TTG GAT CCT ATG TCC CAT AGC AGG CAT CG and 3′ AAT CTA GAG GAT CCC TAC TTT CTT TCT GCT ATT ATC TTT AAA T using Expand High Fidelity PCR System (Roche), the product was digested with BamHI (New England Biolabs) and ligated into pET15b (Novagen) using Rapid DNA Ligation Kit (Roche) to generate pET15b-RAX(S130P). pET15b-RAX and pET15b-RAX(S130P) were transformed into Rosettagami B(DE3) pLysS expression *E. coli* cells (Novagen). These expression cells were grown to A_600_ of 1.0, at which time IPTG (Denville) was added to 1 mM, the cells were incubated shaking at room temperature for one hour. Induced cells were collected by centrifugation, washed in cold PBS, and lysed in Ni-NTA lysis buffer (20 mM Tris-HCl pH 7.5, 500 mM NaCl, 10 mM imidazole, 5 mM 2-mercaptoethanol, 0.1% NP-40, 100 µg/ml lysozyme, 10% glycerol, supplemented with complete protease inhibitor tablet (Roche)). Cells were subjected to one freeze-thaw cycle followed by lysis by sonication. Lysate was clarified by centrifugation for 30 min at 20000×g prior to binding to Ni-NTA superflow resin (Qiagen). Protein-bound resin was poured into a column and attached to an AKTA FPLC, the column was washed to background with wash buffer (20 mM Tris-HCl pH 7.5, 500 mM NaCl, 35 mM imidazole, 10% glycerol), then eluted in a 10 ml linear gradient between wash buffer and elution buffer (20 mM Tris-HCl pH 7.5, 500 mM NaCl, 300 mM imidazole, 10% glycerol). Peak fractions were pooled and dialyzed against dialysis buffer (20 mM Tris-HCl pH 7.5, 500 mM NaCl, 5 mM 2-mercaptoethanol, 10% glycerol).

### RNA isolation, reverse transcription and RT-PCR

RNA was isolated from brains of wild-type, *tm1Gsc* or *rep* mice using TRIZOL (Invitrogen) according to the manufacturer's instructions. RNA was isolated from L929 cell lines using TRIZOL according to the manufacturer's instructions. Following RNA isolation, residual genomic DNA contamination was removed by DNAse I treatment using DNA-free (Ambion). RNA was reverse transcribed using the SuperScript III cDNA First Strand Synthesis Kit (Invitrogen) according to manufacturer's instructions using random hexamer primers. RT-PCR using Clontech Advantage 2 Taq was performed using 18SrRNA primers on DNAse treated RNA (withour reverse transcription) and cDNA samples to determine the efficacy of DNAse treatment. RT-PCR using this same protocol was used to amplify sequences in the 5′ and 3′ regions of RAX, cDNA from 100 ng RNA was used per amplification reaction, and the reaction cycle number titrated for each primer set to determine the logarithmic range for product growth. Cycling was as follows: 1′ 95°C, followed by cycles of 1′ 95°C, 30″ T_m_, 1′ 68°C, finishing with 5′ 68°C. Primer sequences, melting temperatures and cycle numbers were as follows:RAX Exons 2 and 3: 31 cycles, T_m_ = 52°C. 5′ primer TAA GCC TGG GAA AAC ACC, 3′ primer CCA GCT TCT TAC TCG TAC CTT; RAX Exon 8: 30 cycles, T_m_ = 60°C. 5′primer TCT CTT CAG ATT CCG TCA ACT TTC, 3′ primer ACA TTC ATC ACA AGC CTC AAC AC. 18S rRNA: 25 cycles, T_m_ = 55°C. 5′ primer ATT GAC GGA AGG GCA CCA CCA G, 3′ primer CAA ATC GCT CCA CCA ACT AAG AAC G. Realtime PCR was performed using SYBR Green Core reagents (Ambion) using 18S rRNA primers from RT-PCR method and FLAG-PACT/RAX primers: 5′ primer CTA CAA GGA CGA TGA CGA TAA GC, 3′ primer CAG CTT CTT ACT TGT ACC TTC ACC. The reactions were run in a Roche Lightcycler 480, cycling was as follows: 3′ 95°C followed by 50 cycles of 30″ 95°C, 1′ 52°C, 30″ 72°C (SYBR green signal was acquired during the 72°C incubation per cycle).

### DsRNA electrophoretic mobility shift assay

The RNA oligonucleotide GGG AAC AAA AGC UGG GUA CCG GGC CCC CCC was 5′ end labelled using T4 polynucleotide kinase (Promega) according to the manufacturer's instructions. The oligonucleotide GGG GGG GCC CGG UAC CCA GCU UUU GUU CCC was annealed to the radiolabelled oligonucleotide and ethanol precipitated. His-RAX or His-RAX(S130P) was added to binding buffer (20 mM Tris pH 7.5, 50 mM KCl, 2 mM MgCl_2_, 2 mM MnCl_2_, 5% glycerol) to the indicated concentration along with 30000 cpm (25 fmol) labelled dsRNA probe; for poly(I:C) competition, poly(I:C) was added to 10 ng/µl and incubated on ice for 10 minutes prior to adding labelled probe. Binding reactions were incubated at room temperature for 15 minutes, prior to loading on a gel of 0.25× TBE, 5% Acrylamide (37.5∶1 acrylamide∶bis-acrylamide) which was run in cold buffer in a cold room.

### DsRNA pull-down assay

Labelled dsRNA was incubated with His-RAX in binding buffer (20 mM Tris pH 7.5, 50 mM KCl, 2 mM MgCl_2_, 2 mM MnCl_2_, 5% glycerol), the protein was pulled-down by Ni-NTA agarose, washed and bound radioactivity was measured by scintillation counting. Incubation with BSA was used to measure non-specific binding of the probe and unlabelled poly(I:C) was used as a competitor, where indicated.

### Protein isolation and western blot

Protein was isolated from tissue by re-precipitating the organic phase generated during TRIZOL (Invitrogen) RNA isolation according to the manufacturer's instructions. Briefly this involved re-precipitation of protein using isopropanol followed by washing with guanidine hydrochloride. Protein was isolated from cultured cells and whole brain by washing in cold PBS followed by lysis in Triton X-100 lysis buffer (20 mM Tris-HCl pH 7.5, 150 mM NaCl, 1% Triton X-100, 1 mM EDTA, 5 mM 2-mercaptoethanol, 10% glycerol, supplemented with Complete protease inhibitor tablet and PhoSTOP tablet (Roche). Where indicated, sodium arsenite dissolved in water to 100 mM was added to culture media to a final concentration of 100 µM for one hour prior to harvesting cells. Protein was separated using SDS-PAGE and transferred to PVDF membrane for western blotting.

### Antibodies

We used commercial antibodies to PACT/RAX [40], β-actin (Clone AC-15, Sigma-Aldrich, A1978), FLAG (Clone M2, Sigma-Aldrich, Catalog # F1804), P-eIF2α (Ser52) (Invitrogen, Catalog # 44-728), eIF2α (Cell Signalling, Catalog # 9722), His Probe (H-15, Santa Cruz Biotechnology, Catalog # sc-803), PKR (D-20, Santa Cruz Biotechnology, Catalog # sc-708)

### Immunoprecipitation and pulldown

FLAG-RAX was immunoprecipitated from cell lysates with anti-FLAG M2 affinity gel (Sigma). Immunoprecipitation was performed in Triton X-100 lysis buffer. Immunoprecipitated samples were washed twice with lysis buffer then washed once with 1× micrococcal nuclease buffer, resuspended in 50 µl 1× micrococcal nuclease buffer containing 2000 gel units micrococcal nuclease (New England Biolabs), and incubated at 37°C for 30 minutes. Samples were washed an additional two times before separation by SDS-PAGE, and subsequent western blotting. Recombinant 6xHis-tagged WT or mutant RAX (2 µg) were incubated with 200 ng empty vector, FLAG-RAX or FLAG-RAX (S130P) transduced L929 lysate in binding buffer (20 mM Tris-HCl, 150 mM NaCl, 10 mM imidazole, 10% glycerol, supplemented with complete protease inhibitor tablet and PhoSTOP tablet (Roche)), the samples were then pulled-down using Ni-NTA agarose (Qiagen). Two washes were performed with binding buffer, then washed once in 1× micrococcal nuclease buffer, resuspended in 50 µl 1× micrococcal nuclease buffer containing 2000 gel units micrococcal nuclease (New England Biolabs), and incubated at 37°C for 30 minutes. Samples were washed an additional four times with binding buffer before separation by SDS-PAGE, and subsequent western blotting.

### FLAG-RAX decay kinetics

L929 cells expressing empty provirus, FLAG-RAX or FLAG-RAX (S130P) were split into replicate plates and treated with cycloheximide (100 µg/ml). Cells were lysed in Triton X-100 lysis buffer at 0, 2, 4, 8, 12 or 24 hours following cycloheximide treatment. Lysates were analyzed for FLAG-RAX by quantitative western blot using the Odyssey infrared detection system (Licor; IRDye 680 goat anti-rabbit for actin detection and IRDye 800 goat anti-mouse for FLAG detection). FLAG-RAX signal was normalized to actin and the normalized signal was plotted as a function of time.

## Supporting Information

Figure S1 **Landmarks used in morphological analysis of the skull (shown only for left side).** **A** *Landmarks of the skull*: 1. nasale; 2. nasion; 3. bregma; 4. parietal-occipital junction; 5 midline of the interparietal-occipital junction; 6. dorsal midpoint of the foramen magnum; 7. antero-lateral corner of the nasal; 8. parietal-premaxillar-maxillar junction; 9. anterior-most point of the zygomatic spine; 10. posterior-most point of the frontal-maxillary dorsal junction; 11. Anterior-most point of the squamosal-parietal junction; 12. anterior-most point of the temporal-zygomatic junction; 13. ventral-most point of the incisor alveoli; 14. anterior-most point of the anterior palatine foramen; 15. Ventral-most point of the premaxillar, maxillar and anterior palatine foramen junction; 16. posterior-most point of the anterior palatine foramen; 17. medial-most point of the first upper molar cervix; 18. Point of greatest curvature of the posterior margin of molar process; 19. distal-most point of the third upper molar cervix; 20. Posterior-most point of the zygomatic fenetra on the zygomatic process of the squamosal; 21. posterior-most point of the zygomatic/squamosal junction; 22. Antero-medial projection of ectotympanic in basicranial; 23. junction of basioccipital, ectotympanic and basisphenoid; 24. Posterior edge of ectotympanic along its margin with basioccipital; 25. anterior process of auditory bulla; 26. ventral midpoint of the foramen magnum; 27. Anterior-most point of the nasal/premaxillary junction; 28. dorsal-most point of the incisor alveoli; 29; tip of the post-tympanic hook. **B** *Landmarks of the mandible:* 1. tip of the coronoid process; 2. distal-most point of the third lower molar cervix; 3. mesial-most point of the first lower molar cervix; 4. dorsal-most point of the incisor alveoli; 5. inferior-most point of the incisor alveoli; 6. Inferior-most point of the mandibular symphysis; 7. Posterior-most point of insertion site of mandibular transverse muscle; 8. tip of the mandibular process; 9. ventral-most point of the mandibular condyle; 10. anterior-most point of the mandibular condyle; 11. posterior-most point of the mental foramen.
(TIF)
