# Supplementary figures and images for: Missense Mutation in the Second RNA Binding Domain Reveals a Role for Prkra (PACT/RAX) during Skull Development
Source: PLoS One. 2011 Dec 14;6(12):e28537. doi: 10.1371/journal.pone.0028537 (PMC3237451; doi:10.1371/journal.pone.0028537)

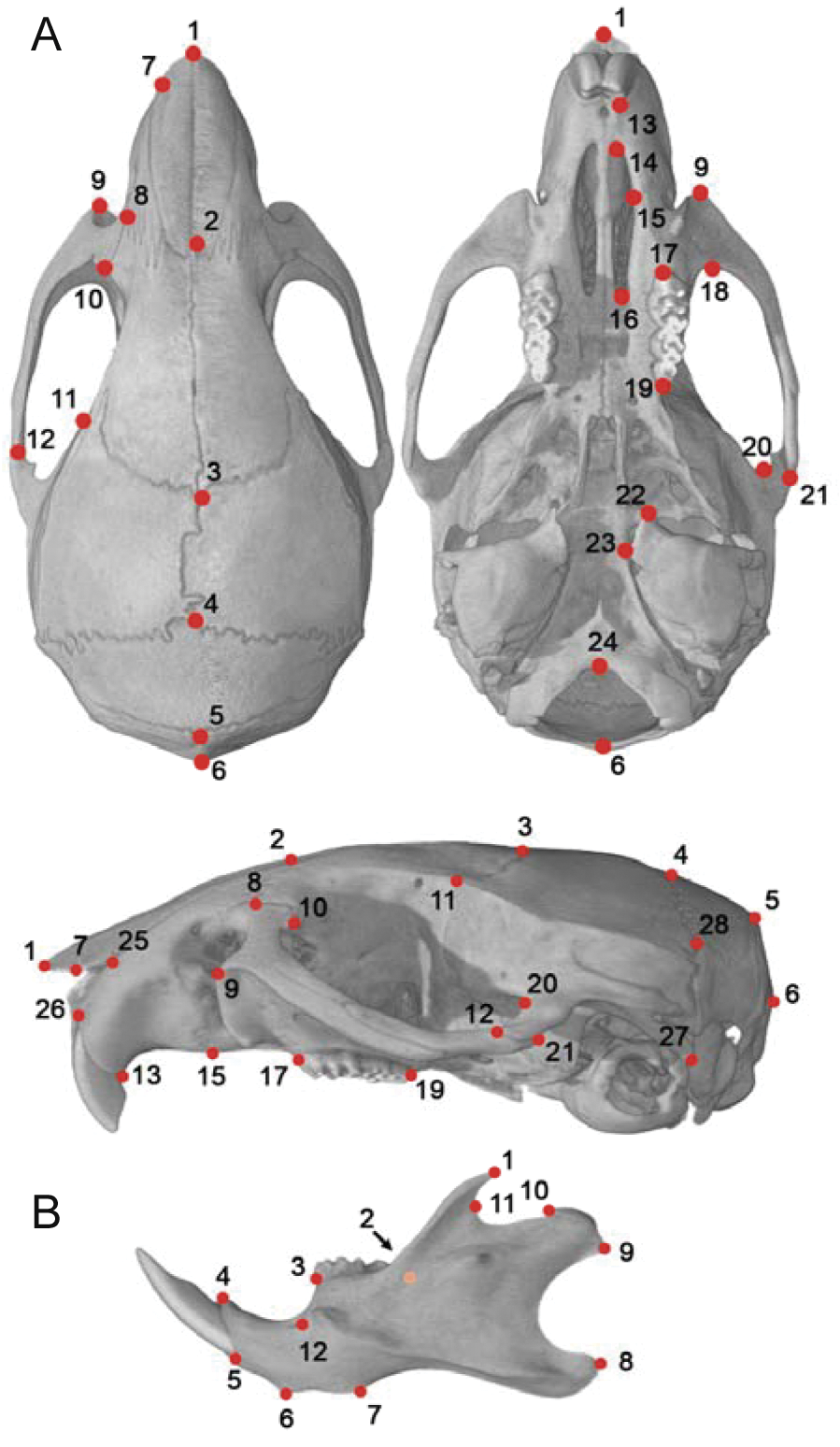

Supplement: Figure S1 — Landmarks used in morphological analysis of the skull (shown only for left side). A Landmarks of the skull: 1. nasale; 2. nasion; 3. bregma; 4. parietal-occipital junction; 5 midline of the interparietal-occipital junction; 6. dorsal midpoint of the foramen magnum; 7. antero-lateral corner of the nasal; 8. parietal-premaxillar-maxillar junction; 9. anterior-most point of the zygomatic spine; 10. posterior-most point of the frontal-maxillary dorsal junction; 11. Anterior-most point of the squamosal-parietal junction; 12. anterior-most point of the temporal-zygomatic junction; 13. ventral-most point of the incisor alveoli; 14. anterior-most point of the anterior palatine foramen; 15. Ventral-most point of the premaxillar, maxillar and anterior palatine foramen junction; 16. posterior-most point of the anterior palatine foramen; 17. medial-most point of the first upper molar cervix; 18. Point of greatest curvature of the posterior margin of molar process; 19. distal-most point of the third upper molar cervix; 20. Posterior-most point of the zygomatic fenetra on the zygomatic process of the squamosal; 21. posterior-most point of the zygomatic/squamosal junction; 22. Antero-medial projection of ectotympanic in basicranial; 23. junction of basioccipital, ectotympanic and basisphenoid; 24. Posterior edge of ectotympanic along its margin with basioccipital; 25. anterior process of auditory bulla; 26. ventral midpoint of the foramen magnum; 27. Anterior-most point of the nasal/premaxillary junction; 28. dorsal-most point of the incisor alveoli; 29; tip of the post-tympanic hook. B Landmarks of the mandible: 1. tip of the coronoid process; 2. distal-most point of the third lower molar cervix; 3. mesial-most point of the first lower molar cervix; 4. dorsal-most point of the incisor alveoli; 5. inferior-most point of the incisor alveoli; 6. Inferior-most point of the mandibular symphysis; 7. Posterior-most point of insertion site of mandibular transverse muscle; 8. tip of the m [file pone.0028537.s001.tif]
